# Unveiling Rare Genetic Variants in DAB2IP: New Insights Into the Pathogenesis of Recurrent Angioedema

**DOI:** 10.1111/all.70184

**Published:** 2025-12-06

**Authors:** Maurizio Margaglione, Maria D'Apolito, Rosa Santacroce, Giorgia Cordisco, Francesco Arcoleo, Tiziana De Pasquale, Ilaria Puxeddu, Andrea Zanichelli, Mauro Cancian

**Affiliations:** ^1^ Medical Genetics, Department of Clinical and Experimental Medicine University of Foggia Foggia Italy; ^2^ UOC di Patologia Clinica e Immunologia, Azienda Ospedaliera Ospedali Riuniti Villa Sofia‐Cervello Palermo Italy; ^3^ Department of Allergology University Hospital “Maggiore Della Carità” of Novara Novara Italy; ^4^ Immuno‐Allergology Unit, Department of Clinical and Experimental Medicine University of Pisa Pisa Italy; ^5^ Department of Biomedical Sciences for Health University of Milan Milan Italy; ^6^ Operative Unit of Medicine, IRCCS Policlinico San Donato Milan Italy; ^7^ Department of Systems Medicine University Hospital of Padua Padua Italy

**Keywords:** angioedema, endotypes, genetics

To the Editor,

It is known that pathogenic variants in the gene encoding the C1‐inhibitor are the most frequent cause of Hereditary angioedema (HAE). In patients with normal C1‐inhibitor, variants in multiple genes were linked to HAE [1]. Recently, a *DAB2IP‐*associated subtype occasionally presenting with hives was identified [2]. DAB2IP is an endogenous inhibitor of VEGF‐receptor‐2 (VEGFR2)‐mediated endothelial signaling. The identification of additional patients carrying DAB2IP gene variants could be of help to better understand how these variants influence observable traits (phenotypes) and establish a clearer genotype–phenotype correlation, revealing the specific role of the gene in disease mechanisms.

We have investigated 279 Italian patients with normal C1‐inhibitor plasma levels and positive or unknown family history of HAE recruited from the Italian Network for Hereditary and Acquired Angioedema (ITACA). All patients had a history of recurrent angioedema without any identifiable variant in HAE‐associated genes despite extensive investigations (Table 1). In five index cases, different *DAB2IP* gene variants were identified (Table S1).

**TABLE 1:** Clinical features of patients carrying DAB2IP gene variants.

#	Sex	Age	Age at the onset of symptoms	Symptoms	*N*°Attacks
1	F	8 years old	8 years old	itching of the lips and skin	10
2	F	74 years old	12 years old	face edema edema of the tongue edema of upper the airways (in one case of the glottis) edema of the gastrointestinal tract	Increase in frequency and severity since 2022
3	F	54 years old	12 years old	subcutaneous edema without urticaria.	1–2/month
4	F	34 years old	12 years old	edema of the lips, cheeks and eyelids	4–5/year
5	M	44 years old	39 years old	edema of the forearm and foot edema of the upper lip	1–2/year

In #1, an in‐frame deletion, c.853_855del (p.K285del), located in exon 6 was detected in the second lysine cluster in the C2 domain, crucial for the binding of DAB2IP to ASK1 (Figure S1) [3]. ASK1 plays a positive role in induced endothelial permeability and the inhibition results in maintaining barrier function. *In silico* analysis predicted a significant effect of the missense p. K285del variation (Table 2).

**TABLE 2:** Information on the different DAB2IP gene variants identified.

Protein variant	Frequencies gnomAD v.4.1	Prediction conservation	Pathogenic prediction	CADD v1.7	Structure prediction
p.K285del	‐exomes:ƒ = 0.0000171 ‐genomes:ƒ = 0.0000131	Very high (0.99)	Mutation Taster: disease causing (0,999)	Quite likely deleterious (20.7)	Pathogenic moderate
p.R590C	‐exomes:ƒ = 0.0000137—genomes: not found	Very high (0.98)	SIFT: Deleterious (0) PolyPhen: Possible damaging (0.68) Mutation Taster: disease causing (0.999)	Deleterious (32)	P18 pocket containing p.R590C
p.R907W	‐exomes:ƒ = 0.00886 ‐genomes:ƒ = 0.00591	Very high (0.95)	SIFT Deleterious (0) PolyPhen: Probably damaging (0.997) Mutation Taster: disease causing (0.998)	Quite likely deleterious (23.3)	(ΔΔG Stability) 0.52 kcal/mol *Destabilising*
p.R966H	‐exomes:ƒ = 0.0004261 ‐genomes: ƒ = 0.003838	High (0.88)	SIFT: Deleterious (0) PolyPhen: Probably damaging (0.975) Mutation Taster: disease causing (0.999)	Probably deleterious (28.5)	Reduced phosphorylation at residues S964 and S971
p.R984W	‐exomes:ƒ = 0.000154 ‐genomes:ƒ = 0.0000526	High (0.87)	SIFT: Deleterious (0) PolyPhen: Probably damaging (0.965) Mutation Taster: disease causing (0.999)	Deleterious (31)	(ΔΔG Stability) 0.46 kcal/mol *Destabilising* P48 pocket containing p.R984W

*Note:* For each genetic variant, the following information is shown: population data obtained from the Genome Aggregation Database (gnomAD; https://gnomad.broadinstitute.org/); evolutionary conservation in the protein sequence using the ScoreCons algorithm (https://www.ebi.ac.uk/thornton‐srv/databases/cgi‐bin/valdar/scorecons_server.pl); pathogenicity prediction evaluated using *in silico* tools, such as Mutation Taster (http://www.mutationtaster.org), Polyphen2 (http://genetics.bwh.harvard.edu/pph2) and SIFT (http://sift.jcvi.org); combined *in silico* prediction of pathogenicity using CADD v1.7 (combined annotation‐dependent depletion) tool (http://cadd.gs.washington.edu/home); putative pathogenic effect on the protein structure using DynaMut2 (https://biosig.lab.uq.edu.au/dynamuth2); putative pathogenic effect on phosphorylation using MusiteDeep (https://github.com/duolinwang/MusiteDeep) and NetPhos‐3.1 (https://services.healthtech.dtu.dk/services/NetPhos‐3.1/).

In #2, a heterozygous variant in exon 10 was identified, c.1768C > T (p.R590C). The residue R590 is predicted to be in the binding pocket P18, which may affect the affinity to ligands and produce an important effect in binding capabilities. The substitution disturbs bond formation with nearby residues, with N587 and E594 (Figure S2).

In #3, a heterozygous variant in exon 12 was identified, c.2719C > T (p.R907W). The residue R907 is in a region crucial for binding to AKT1 aa (646–943) [4]. *In silico* analysis predicted a significant effect of the missense p.R907W variation on protein stability (Table 2).

In #4, a heterozygous variant in exon 12 at nucleotide was identified, c.2897G > A (p.R966H). The amino acid residue R966 lies in a disordered region (residues #895–998) that contains a series of Serines that are predicted to undergo post‐translational modification through phosphorylation. *In silico* analysis predicted a significant effect on the phosphorylation of Serines at residues #964 and #971 (Table 2).

In #5, a heterozygous variant in exon 12 of the DAB2IP was identified, c.2950C > T (p.R984W). The substitution may disturb bond formation with nearby residues, V982 and K986 (Figure S2). Protein modelling analysis predicted a significant effect of the missense p.R984W variation (Table 2).

Present findings emphasize that DAB2IP functions as an endogenous inhibitor of stimuli inducing endothelial permeability by inhibiting the VEGFR2‐dependent vasogenic signaling [2]. This evidence further stresses the central role of VEGF‐mediated signaling in the regulation of endothelial permeability [5]. At present, there are no data available to suggest a genotype–phenotype correlation.

None of the patients carrying a *DAB2IP* gene variant showed a positive family history. One of the patients was adopted. Another belonged to a foreign family and it was difficult to make an accurate assessment of the patient's family history. This represents a limitation of the study. Patients with recurrent angioedema and a negative family history, and normal values of C1‐INH, who are unresponsive to antihistamines, omalizumab, or ciclosporine, are an important diagnostic and therapeutic challenge in daily practice. A proportion of patients initially diagnosed as idiopathic non‐histaminergic acquired angioedema was diagnosed as HAE even in the absence of a family history [6]. The reduced penetrance described in some HAE sub‐settings (i.e., HAE‐FXII) and the loss of contact of patients with other relatives may well explain this discrepancy. Screening for pathogenic variants in genes associated with HAE would be of value in patients with angioedema of uncertain etiopathogenesis.

Finally, it is remarkable that none of the patients with a *DAB2IP* gene variant showed hives, suggesting that loss‐of‐function variants predispose to HAE rather than familial urticaria.

In conclusion, loss of function DAB2IP gene variants can induce an imbalance in signalling pathways, such as that VEGFR2‐dependent, leading to angioedema attacks. Further studies are needed to establish whether variants affecting the VEGF‐VEGFR2 axis influence the regulation of the bradykinin pathway.

## Author Contributions

Design and conception of the study: Maurizio Margaglione, Maria D'Apolito, Andrea Zanichelli, Mauro Cancian. Clinical investigation: Francesco Arcoleo, Tiziana De Pasquale, Ilaria Puxeddu. Methodology and analysis of the data: Maurizio Margaglione, Maria D'Apolito, Rosa Santacroce, Giorgia Cordisco. Drafting of the manuscript: Maurizio Margaglione, Maria D'Apolito, Mauro Cancian. All authors contributed to data collection and interpretation, and reviewed and edited the work.

## Funding

The authors received no specific funding for this work.

## Conflicts of Interest

The authors declare no conflicts of interest.

## Supporting information


**Table S1:**List of *DAB2IP* gene variants identified in the study cohort.
**Figure S1:** Schematic diagram of DAB2IP domains and localization of identified *DAB2IP* gene variations. DAB2IP functional domains interact with ASK1, GSK3β, VEGFR2, and PP2A (C2); RasGTP (GAP); AKT (PER); PI3K p85 subunit (PR). Protein domain boundaries are from UniProt (https://www.uniprot.org/uniprotkb/Q5VWQ8/entry), except for PER (ref 33; Fig.S3) and LZ (https://2zip.molgen.mpg.de/). PH: pleckstrin homology domain; C2: protein kinase C‐conserved domain 2; GAP: GTPase‐activating protein domain; PER: period‐like domain; PR: proline‐rich domain; LZ: leucine‐zipper domain.
**Figure S2:** Effects of DAB2IP variants on protein structure as predicted using the DynaMut2 online server. The normal and the mutated amino acid at #590 (upper panel: A and B, respectively) and #984 (lower panel: C and D, respectively) are shown. Types of bonds forming among residues are shown (legend at the bottom). Arrows indicate bonds differing between the normal and the variant molecule.

## Data Availability

The data that support the findings of this study are available from the corresponding author upon reasonable request.
