# Supplementary material for: Unveiling Rare Genetic Variants in DAB2IP: New Insights Into the Pathogenesis of Recurrent Angioedema
Source: Allergy. 2025 Dec 6;81(7):2519–22. doi: 10.1111/all.70184 (PMC13342756; doi:10.1111/all.70184)
Supplement: Supplementary file 1 — Table S1:List of DAB2IP gene variants identified in the study cohort. Figure S1: Schematic diagram of DAB2IP domains and localization of identified DAB2IP gene variations. DAB2IP functional domains interact with ASK1, GSK3β, VEGFR2, and PP2A (C2); RasGTP (GAP); AKT (PER); PI3K p85 subunit (PR). Protein domain boundaries are from UniProt (https://www.uniprot.org/uniprotkb/Q5VWQ8/entry), except for PER (ref 33; Fig.S3) and LZ (https://2zip.molgen.mpg.de/). PH: pleckstrin homology domain; C2: protein kinase C‐conserved domain 2; GAP: GTPase‐activating protein domain; PER: period‐like domain; PR: proline‐rich domain; LZ: leucine‐zipper domain. Figure S2: Effects of DAB2IP variants on protein structure as predicted using the DynaMut2 online server. The normal and the mutated amino acid at #590 (upper panel: A and B, respectively) and #984 (lower panel: C and D, respectively) are shown. Types of bonds forming among residues are shown (legend at the bottom). Arrows indicate bonds differing between the normal and the variant molecule. [file ALL-81-2519-s001.docx]

**Materials and Methods**

*Patient Enrolment and DNA Extraction*

A total of 279 patients belonging to independent families and additional unrelated index patients with recurrent angioedema without wheals were recruited from ITACA Centres and enrolled for the study from April 2021 to January 2025. All patients presented with angioedema symptoms and fulfilled the following criteria: (1) recurrent swelling of the skin or mucous membranes, abdominal pain attacks, tongue swelling, or laryngeal attacks; (2) no association with urticaria documented in the medical history; (3) unresponsiveness to antihistamines, omalizumab, or cyclosporine; (4) normal C1 INH function and concentration in plasma; and (5) angioedema that was not a relapsing acute reaction to allergens, drugs, or infections. Clinical and family history of all participants was evaluated. All enrolled subjects (legal guardians for minors) underwent pre-test counselling during which they were informed about the significance of molecular analysis, provided information about their personal and familial history, and gave written informed consent for the anonymous use of their clinical data. After approval of local ethics committees, the study was carried out according to the principles of the Declaration of Helsinki.

*Molecular Analysis*

Molecular testing was carried out by analyzing a panel of target genes through a next generation sequencing (NGS)-based procedure. In particular, a custom panel was used that included genes previously associated with HAE (*SERPING1, F12, ANGPT1, PLG, KNG1, MYOF, HS3ST6, and DAB2IP*). For each gene, we analyzed the coding regions, 50 bp in each of the intronic boundaries, the promoter, and the 3′UTR. HaloPlex technology (Agilent, Santa Clara, CA, USA) was used for library preparation. A total of 1.8 ng of genomic(g)DNA was processed through the HaloPlex Target Enrichment System (Agilent Technologies, Santa Clara, CA, USA) for Illumina multiplexed sequencing. Briefly, gDNA was enzymatically fragmented using a pool of restriction enzymes. The obtained fragments were enriched with hybridization with the custom capture probes, and then purified and PCR-amplified to obtain a DNA library of sample. During this process, each genomic DNA sample was univocally tagged with a barcode sequence to allow for sample multiplexing during the subsequent sequencing step using the MiSeq (Illumina, San Diego, CA, USA) instrument using a Illumina Cardridge 300v2 Standard, running 16 samples for each sequencing run to obtain an average coverage of about 200X.

The Alissa Align & Call v1.0.2.10 tool (Agilent Technologies, Santa Clara, CA, USA) was used to perform sequencing analysis using the genome build hg38 as a reference, to carry out alignments, variant calling, and quality filtering. Variant filtering and interpretation were done using Alissa Interpret v5.2.6 CE IVD software (Agilent Technologies, Santa Clara, CA, USA. This tool allowed to acquire a list of genomic variants that can be prioritized using a customizable pipeline in order to highlight pathogenic mutations or potentially pathogenic variants. Pathogenic variants or of doubtful significance were confirmed with standard Sanger sequencing on an automated analyser, SeqStudio (Thermo Fisher Scientific, Waltham, MA, USA). The *DAB2IP* gene (NM_001395010.1) exons 6, 10, and 12 were amplified using specific primers and PCR products were then sequenced using BigDye Terminator v.3.1 (Thermo Fisher Scientific, Waltham, MA, USA), according to standard protocols. [1] The NCBI GenBank accession numbers NM_001395010.1 (Ensembl: ENST00000408936.8) and UniProt ID: Q5VWQ8-1 were used as reference sequences for DAB2IP nucleotide and protein sequences, respectively.

*In silico* *analysis of pathogenicity*

Variant classification was based on their position into the gene structure (exonic or intronic), population frequency, coding effect, ClinVar classification, and functional predictions. To predict the functional impact of variants, computational tools were used. *In silico* possible disease-causing missense variants were evaluated using prediction tools such as Mutation Taster (http://www.mutationtaster.org), Polyphen2 (http://genetics.bwh.harvard.edu/pph2) and SIFT (http://sift.jcvi.org) according the latest recommendations (2022) for PP3/BP4 rules. The CADD v1.7 (combined annotation-dependent depletion) tool (http://cadd.gs.washington.edu/home) [2] was used to integrate multiple annotations for the deleteriousness of single nucleotide variants, insertion/deletion variants in the human genome. Scores were relative to all other scores. They were log10 scaled with higher values representing a more deleterious variant consequence. (<15.0 = likely benign, 15.0-19.9 = potentially deleterious, 20.0-24.9 = quite likely deleterious, 25.0-29.9 = probably deleterious, >29.9 = highly likely deleterious). Population data were obtained from the Genome Aggregation Database (gnomAD; https://gnomad.broadinstitute.org/). Evolutionary conservation in the sequence was established based on UniRef90 sequence alignments using the ScoreCons algorithm (https://www.ebi.ac.uk/thornton-srv/databases/cgi-bin/valdar/scorecons_server.pl). [3] Score is allowed to range from 0 (no conservation) to 1 (complete conservation). VarSome (https://varsome.com accessed on 15 February 2025), ClinVar (https://www.ncbi.nlm.nih.gov/clinvar accessed on 15 February 2025), and Franklin by genoox (https://franklin.genoox.com/clinical-db/home on accessed on 15 February 2025), ProtVar (https://www.ebi.ac.uk/ProtVar on accessed on 15 February 2025) were used as tools to sum up actual knowledge about the variants. DAB2IP AlphaFold structure prediction model (AF-Q5VWQ8-F1-model_v4.pdb) was then used as a template to study the putative pathogenic effect of the substitution using DynaMut2 (https://biosig.lab.uq.edu.au/dynamuth2), a web server, which can be used to analyze and visualize protein dynamics by sampling conformations and assess the impact of mutations on protein dynamics and stability resulting from vibrational entropy changes. In this tool, ΔΔG ≥ 0 is considered stabilizing, and ΔΔG < 0 is considered destabilizing. [4] DAB2IP phosphorylation prediction tools as MusiteDeep (https://github.com/duolinwang/MusiteDeep) and NetPhos-3.1 (https://services.healthtech.dtu.dk/services/NetPhos-3.1/) were used to analyze the effect of mutations on post-translational protein modifications.

**References**

1. D'Andrea G, Colaizzo D, Vecchione G, Grandone E, Di Minno G, Margaglione M; GLAnzmann's Thrombasthenia Italian Team (GLATIT). Glanzmann's thrombasthenia: identification of 19 new mutations in 30 patients. Thromb Haemost. 2002;87:1034-42.
2. Rentzsch P, Witten D, Cooper GM, Shendure J, Kircher M. CADD: predicting the deleteriousness of variants throughout the human genome. Nucleic Acids Res. 2019 Jan 8;47(D1):D886-D894.
3. Valdar WS, Thornton JM. Protein-protein interfaces: analysis of amino acid conservation in homodimers. Proteins. 2001 Jan 1;42(1):108-24.
4. Rodrigues CHM, Pires DEV, Ascher DB. DynaMut2: Assessing changes in stability and flexibility upon single and multiple point missense mutations. Protein Sci. 2021 Jan;30(1):60-69.

**Table S1.** List of *DAB2IP* gene variants identified in the study cohort.

| # | Reference SNP ID | Variant type | Genomic | HGVS* Coding (cDNA) | HGVS *  Protein Level | IUPAC Protein |
| --- | --- | --- | --- | --- | --- | --- |
| 1 | rs756722949 | in frame deletion | chr9-121760109_111 | c.853_855del | p.(Lys285del) | p.K285del |
| 2 | rs766959002 | missense | chr9-121768502 | c.1768C>T | p.(Arg590Cys) | p.R590C |
| 3 | rs56200518 | missense | chr9-121773247 | c.2719C>T | p.(Arg907Trp) | p.R907W |
| 4 | rs371407818 | missense | chr9-121773424 | c.2896C>T | p.Arg(966Cys) | p.R966C |
| 5 | rs774366709 | missense | chr9-121773478 | c.2950C>T | p.(Arg984Trp) | p.R984W |

For each genetic variant, the following information is shown: reference singe-nucleotide polymorphism (SNP) ID number (rs), Variant Type, variant nomenclature at DNA and protein level according to Human Genome Variation Society (* HGVS) guidelines, variant protein IUPAC nomenclature. The NCBI GenBank accession numbers NM_001395010.1 (Ensembl: ENST00000408936.8) and UniProt ID: Q5VWQ8-1 were used as reference sequences for DAB2IP nucleotide and protein sequences.

**Legend to the Figures**

**Figure S1.** Schematic diagram of DAB2IP domains and localization of identified *DAB2IP* gene variations. DAB2IP functional domains interact with ASK1, GSK3β, VEGFR2, and PP2A (C2); RasGTP (GAP); AKT (PER); PI3K p85 subunit (PR). Protein domain boundaries are from UniProt (https://www.uniprot.org/uniprotkb/Q5VWQ8/entry), except for PER (ref 33; Fig.S3) and LZ (https://2zip.molgen.mpg.de/). PH: pleckstrin homology domain; C2: protein kinase C-conserved domain 2; GAP: GTPase-activating protein domain; PER: period-like domain; PR: proline-rich domain; LZ: leucine-zipper domain.

**Figure S2.** Effects of DAB2IP variants on protein structure as predicted using the DynaMut2 online server. The normal and the mutated amino acid at #590 (upper panel: A and B, respectively) and #984 (lower panel: C and D, respectively) are shown. Types of bonds forming among residues are shown (legend at the bottom). Arrows indicate bonds differing between the normal and the variant molecule.

**Figure S1**


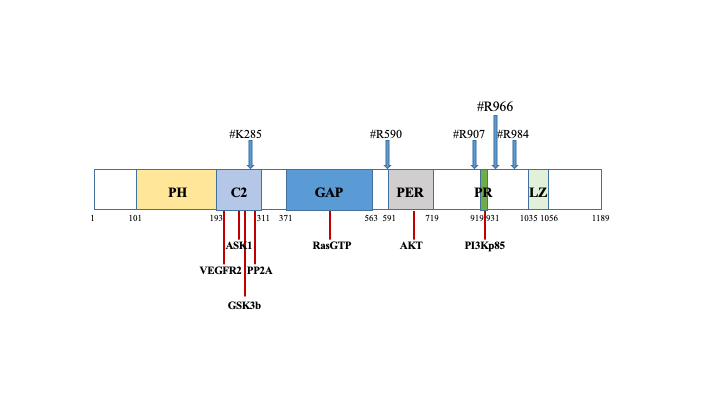


**
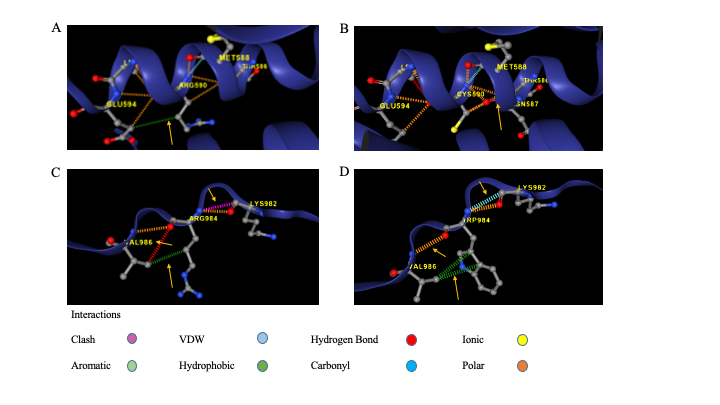
Figure S2**
